# MicroRNA-106a Inhibits Autophagy Process and Antimicrobial Responses by Targeting ULK1, ATG7, and ATG16L1 During Mycobacterial Infection

**DOI:** 10.3389/fimmu.2020.610021

**Published:** 2021-01-05

**Authors:** Kunmei Liu, Dantong Hong, Fan Zhang, Xin Li, Meng He, Xuebo Han, Guolin Zhang, Guangxian Xu, Nicola J. Stonehouse, Zhongjia Jiang, Weijun An, Le Guo

**Affiliations:** ^1^Ningxia Key Laboratory of Clinical and Pathogenic Microbiology, General Hospital of Ningxia Medical University, Yinchuan, China; ^2^Ningxia Key Laboratory of Cerebrocranial Diseases, Ningxia Medical University, Yinchuan, China; ^3^Department of Medical Laboratory, School of Clinical Medicine, Ningxia Medical University, Yinchuan, China; ^4^Department of Orthopaedics, General Hospital of Ningxia Medical University, Yinchuan, China; ^5^Suzhou Institute for Drug Control, Suzhou, China; ^6^School of Molecular and Cellular Biology, Faculty of Biological Sciences, University of Leeds, Leeds, United Kingdom

**Keywords:** *Mycobacterium tuberculosis*, autophagy, miR-106a, ULK1, ATG7, ATG16L1

## Abstract

Autophagy is a key element of innate immune response against invading pathogens including *Mycobacterium tuberculosis* (*M. tuberculosis*). The emerging roles of microRNAs in regulating host antimicrobial responses against *M. tuberculosis* have gained widespread attention. However, the process by which miRNAs specifically influence antibacterial autophagy during mycobacterial infection is largely uncharacterized. In this study, we demonstrate a novel role of miR-106a in regulating macrophage autophagy against *M. tuberculosis*. H37Ra infection leads to downregulation of miR-106a in a time- and dose-dependent manner and concomitant upregulation of its three targets (ULK1, ATG7, and ATG16L1) in THP-1 macrophages. MiR-106a could inhibit autophagy activation and antimicrobial responses to *M. tuberculosis* by targeting ULK1, ATG7, and ATG16L1. Overexpression of miR-106a dramatically inhibited H37Ra-induced activation of autophagy in human THP-1 macrophages, whereas inhibitors of miR-106a remarkably promoted H37Ra-induced autophagy. The inhibitory effect of miR-106a on autophagy process during mycobacterial infection was also confirmed by Transmission Electron Microscope (TEM) observation. More importantly, forced expression of miR-106a increased mycobacterial survival, while transfection with miR-106a inhibitors attenuated the survival of intracellular mycobacteria. Taken together, these data demonstrated that miR-106a functioned as a negative regulator in autophagy and antimicrobial effects by targeting ULK1, ATG7, and ATG16L1 during *M. tuberculosis* infection, which may provide a potential target for developing diagnostic reagents or antibacterials against tuberculosis.

## Introduction

Tuberculosis (TB) is a communicable disease that is one of the top 10 causes of death worldwide and the leading cause of death from a single infectious agent (1). *Mycobacterium tuberculosis* (*M. tuberculosis*) infects approximately one third of the global population, making *M. tuberculosis* the leading bacterial cause of death in humans worldwide (2). However, only about 10% of individuals infected with *M. tuberculosis* develop active TB, while the majority of cases, about 90%, exhibit latent infection, suggesting a crucial role for host innate immunity in controlling *M. tuberculosis* infection (3). As the first line of immune defense against *M. tuberculosis*, macrophages not only recognize *M. tuberculosis* by pattern recognition receptors (PRRs), but also present bacterial peptide from *M. tuberculosis* to T lymphocytes, thus resulting in the activation of adaptive immune responses against *M. tuberculosis* (4). Moreover, the activation of antibacterial autophagy through ubiquitination of *M. tuberculosis* promotes the innate immune response against *M. tuberculosis* infection (5). Upon infection by *M. tuberculosis*, macrophages can launch a variety of innate immune defenses against *M. tuberculosis* (6, 7). In contrast, *M. tuberculosis* utilizes many strategies to evade host defense response for surviving and persisting within human macrophages (8). For instance, *M. tuberculosis* can arrest normal phagosome maturation, and avoid fusion with lysosomes to escape degradation by lysosomal hydrolases (9, 10).

Autophagy is widely recognized as a cellular process that can encapsulate macromolecules, organelles, or intracellular pathogens in double membrane-layered vesicles and deliver them to lysosomes for degradation (11). A number of autophagy-related genes (ATGs) orchestrate signaling events that regulate autophagy flux including formation of phagophore, autophagosome formation and phagolysosomal maturation during microbial invasion (12, 13). Among the ATGs, ULK1, ATG7, and ATG16L1 are essential for autophagy. ULK1 is a key component in the ULK1 complex which is crucial for initiation and formation of autophagosome (14). ATG7 has dual functions in autophagy regulation. First, ATG7 is essential for formation of a functional autophagosome by conjugating ATG5 to ATG12 as an E1-like enzyme. Second, ATG7 conjugates LC3-I to phosphatidylethanolamine, forming a mature autophagosomal membrane protein, LC3-II (15). Moreover, ATG16L1 is a component of the ATG12–ATG5–ATG16L1 complex, which localizes to phagophore membranes and stimulates the transfer of LC3 from ATG3 to PE (16).

MicroRNAs (miRNAs) are a growing family of small non-coding RNAs that function as post-transcriptional regulators of gene expression by targeting mRNAs for translational repression or cleavage (17). Additionally, miRNAs have been proven to be involved in a variety of biological pathways, including development, homeostasis and diseases (18, 19). A growing body of evidence suggests that miRNAs also play important roles in regulating autophagy, especially in tumors (20, 21). However, the potential roles of miRNAs in regulating autophagy process during *M. tuberculosis* infection need to be further explored. In this study, we characterized the potential role of miR-106a in modulating autophagy process and affecting bacterial clearance in macrophages. Our study demonstrated that miR-106a expression was significantly decreased after mycobacterial infection in human THP-1 macrophages. Downexpression of miR-106a increased the expression levels of ULK1, ATG7, and ATG16L1 and promoted formation of autophagosomes in human THP-1 macrophages, thus attenuating bacterial survival. However, forced expression of miR-106a had the opposite effect. These findings provide a better understanding of miRNAs on regulating innate immunity and host defense against *M. tuberculosis*.

## Materials and Methods

### Selection of Microarray Datasets and Analysis

The miRNA microarray dataset (GSE119494) was selected for analysis. GSE119494 contains miRNA expression data from PBMCs of three healthy donors and three active pulmonary tuberculosis (TB) patients. The miRNA expression profiling file was obtained, and the expression data of miR-17 family (miR-17, miR-20a, miR-20b, miR-106a, miR-106b, and miR-93) was selected for analysis. The data of miR-17 family were mean centered and represented by a heat map using Multi Experiment Viewer software (MeV).

### Cells and Bacterial Culture

The human monocyte/macrophage cell line THP-1, human embryonic kidney 293T cells (HEK 293T), *Mycobacterium bovis* BCG and *M. tuberculosis* H37Ra were obtained from the American Type Culture Collection (ATCC). The THP-1 cells were cultured in suspension using RPMI1640 (GIBCO) supplemented with 10% fetal bovine serum and gentamycin. THP-1 cells were differentiated into adherent, well-spread macrophages with 100 nM phorbol 12-myristate 13-acetate (PMA, Sigma) to the well and maintenance for 3 days. BCG or H37Ra was grown in Middlebrook 7H9 broth medium (Goybio, China) supplemented with albumin dextrose catalase supplement.

### Cell *T*ransfections and Chemical Reagent Treatment

The miR-106a mimics, miR-106a inhibitor, ATG7 siRNA, ATG16L1 siRNA, and ULK1 siRNA were purchased from GenePharma biotechnology company. To assay luciferase activity, HEK 293T cells were cotransfected with the pmirGLO luciferase constructs (WT or Mut) and miR-106a mimics or miR-106a inhibitor using Lipofectamine 2000 according to the manufacturer’s instruction. For autophagy analysis, THP-1 macrophages were transfected with 50 nM mimic negative control (mimic nc) or miR-106a mimics; inhibitor negative control (inhibitor nc) or miR-106a inhibitor; 50 pmol ATG7 siRNA, ATG16L1 siRNA, or ULK1 siRNA according to the manufacturer’s instructions. Several chemical reagents were also used to treat THP-1 macrophages: a lysosome inhibitor, bafilomycin A1 (100 nM; Baf A1, Selleck); An autophagy inducer, rapamycin (50 μg/ml; Rapa, Solarbio Science & Technology Co.).

### RNA Preparation, Real-Time PCR, and Western Blotting

For quantitative real-time PCR (RT-PCR) analysis, total RNA from cells was isolated using RNA simple Total RNA Kit (Tiangen Biotech), and miRNAs were performed using the miRcute miRNA isolation kit (Tiangen Biotech) according to the manufacturer’s instructions. RT-PCR was performed using Hairpin-it™ miRNAs RT-PCR Quantitation Kit (GenePharma, China) and samples were amplified for 40 cycles as follows: 95°C for 12 s, 62°C for 40 s, and 72°C for 30 s. The miR-106a expression was calculated relative to U6 snRNA. For Western blotting, proteins were loaded onto 12 or 15% SDS-PAGE gels and transferred to a polyvinylidene difluoride membrane (PVDF). Membranes were blocked in 5% non-fat milk in PBST for 1 h, and incubated with anti-ULK1(Abcam, ab167139), anti-ATG16L1 (Abcam, ab188642), anti-ATG7 (Abcam, ab52472), anti-LC3 (Abcam, ab51520), and anti-GAPDH (Abcam, ab245355). Immunoreactive band was performed using ECL reagent (Amersham Pharmacia) and quantified by using Image J software (NIH).

### Bioinformatics Analysis, Plasmid Constructs, and Luciferase Assay

To perform miRNA profiling assays, we downloaded a miRNA expression dataset (GSE119494). The raw data are available on the Gene Expression Omnibus website (http://www.ncbi.nlm.nih.gov/geo/). The heatmap was analyzed by using Multiple Experiment Viewer version 4.9.0. miRNA targets were performed using miRanda (http://www.microrna.org) and TargetScan (http://www.targetscan.org). About 500 bp 3′-UTR fragments from ULK1, ATG16L1, or ATG7, containing the miR-106a-binding elements, were produced by PCR and were inserted into the pmirGLO dual-luciferase reporter vector (Promega). Mutant derivatives of the construct were also inserted into the pmirGLO dual-luciferase reporter vector (Promega). The HEK 293T cells were cultured into a 12-well plate and cotransfected with the luciferase constructs (WT or Mut) together with the miR-106a mimics or miR-106a inhibitors, respectively. Luciferase assays were performed at 24 h after transfection using the Dual-Luciferase Reporter Assay Kit (TransGen Biotech, Beijing).

### Immunofluorescence Staining and Confocal Microscopy Analysis

The THP-1 macrophages were fixed with 4% paraformaldehyde (Sigma) followed by permeabilization with 0.2% Triton X-100 (Thermo Fisher Scientific). Cells were blocked with 3% BSA and labelled with Rabbit polyclonal to LC3 antibody (Abcam, ab51520) and visualized by Alexa Fluor 488-conjugated Affinipure Goat Anti-Rabbit IgG (Proteintech). Nuclei were stained with DAPI. The fluorescence images of cells were acquired and examined using a confocal microscope (Olympus, Japan). To quantify autophagy, the number of LC3 punctate dots was calculated by ImageJ Software (Version 1.49). At least 10 cells per experimental group were counted and each condition was assayed in triplicate.

### Transmission Electron Microscopy

The THP-1 macrophages were collected and fixed in 2% glutaraldehyde, and then postfixed with 1% OsO4 for 2 h. After dehydration in a graded series of ethanol, the samples were transferred to propylene oxide and embedded in Epon. Ultrathin sections, about 80 nm thick, were cut and stained with uranyl acetate and lead citrate. Imaging was performed by a transmission electron microscopy (TEM, Hitachi H-7650). For each sample, group, 15 cellular cross-sections were counted.

### Colony-Forming Unit Assay

To assess bacterial viability within human THP-1 macrophages, Colony-Forming Unit (CFU) assay was performed. Briefly, the THP-1 macrophages were transfected with miR-106a mimic, miR-106a inhibitor, mimic nc or inhibitor nc for 24 h, or treated with rapamycin plus miR-106a for 24 h. Moreover, the THP-1 macrophages were also transfected with miR-106a mimic in the presence of rapamycin (50 μg/ml) for 24 h. The cells were infected with H37Ra at a MOI of 10 for 3 h, and then washed with PBS to remove extracellular H37Ra. After that, the infected cells were cultured for an additional 24 h. Quantitative culturing was performed using 10-fold serial dilutions on Middlebrook 7H10 agar plates. Plates were incubated for 2 weeks, and colonies on plates were counted.

### Statistical Analysis

The results are represented as mean ± SD of independent experiments. Statistical analyses were performed using two-tailed Student’s *t*-test. Significant differences were assigned to p values <0.05, <0.01 and <0.001, denoted by *, **, and ***, respectively.

## Results

### miR-106a Expression in Human Macrophages After Mycobacterial Infection

**Figure 1:**
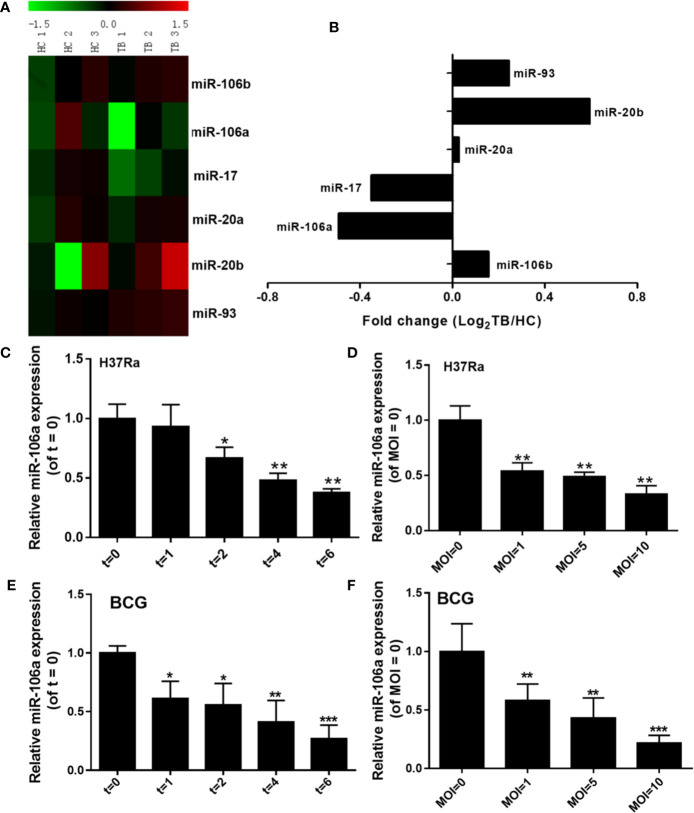
miR-106a is reduced after mycobacterial infection *in vitro*. **(A)** Heatmap analysis shows downregulated (green) and upregulated (red) miRNAs in miR-17 family from tuberculosis (TB) patients and healthy controls (HCs) in the GEO public databases (GSE119494). **(B)** Expression levels of miR-17 family miRNAs in TB patients and HCs from the GEO public databases (GSE119494). Fold change was calculated by dividing the average signal intensity of TB patients by that of HCs. **(C)** The differentiated THP-1 macrophages were infected with H37Ra at a MOI of 10 for the indicated time points, and miR-106a expression was subsequently determined using qRT-PCR. The miR-106a expression levels are indicated relative to expression at 0 h. **(D)** The differentiated THP-1 macrophages were infected with H37Ra at indicated MOIs for 24 h. The miR-106a expression levels are indicated relative to expression without H37Ra infection. **(E)** The differentiated THP-1 macrophages were infected with BCG (MOI of 10) for the indicated time points, and miR-106a expression was subsequently determined using qRT-PCR. The miR-106a expression levels are indicated relative to expression at 0 h. **(F)** The differentiated THP-1 macrophages were infected with BCG at indicated MOIs for 24 h. The miR-106a expression levels are indicated relative to expression without BCG infection. All data above represent the means ± SD from at least three independent experiments. *p < 0.05, **p < 0.01, ***p < 0.001.

To evaluate the expression profiles of miR-17 family miRNAs in peripheral blood mononuclear cells (PBMCs) from patients with active pulmonary tuberculosis (TB), we analyzed miRNA microarray datasets (GSE119494) from the Gene Expression Omnibus (GEO) public database. The heatmap revealed that miR-106a and miR-17 showed the great magnitude of downregulation among the miR-17 family miRNAs (**Figure 1A**). In addition, we compared the expression of miR-106a and mir-17 in PBMCs from active pulmonary tuberculosis (TB) patients and healthy controls (HCs). The expression levels of miR-106a and miR-17 were significantly lower in PBMCs from active pulmonary TB patients than in HCs (**Figure 1B**). Previous studies have showed that miR-17 was downregulated in macrophages and regulated autophagy by targeting Mcl-1 and STAT3 during mycobacterial infection (22). As miR-106a’s functional role in regulation of *M. tuberculosis* infection remains uncharacterized, we choose miR-106a for our further study. We used *M. tuberculosis* H37Ra or *Mycobacterium bovis* BCG to infect the differentiated THP-1 macrophages, and found that both H37Ra (**Figures 1C, D**) and BCG (**Figures 1E, F**) strains could significantly reduce miR-106a expression in a time- and dose-dependent manner.

### miR-106a Directly Targets ULK1, ATG7, and ATG16L1

**Figure 2:**
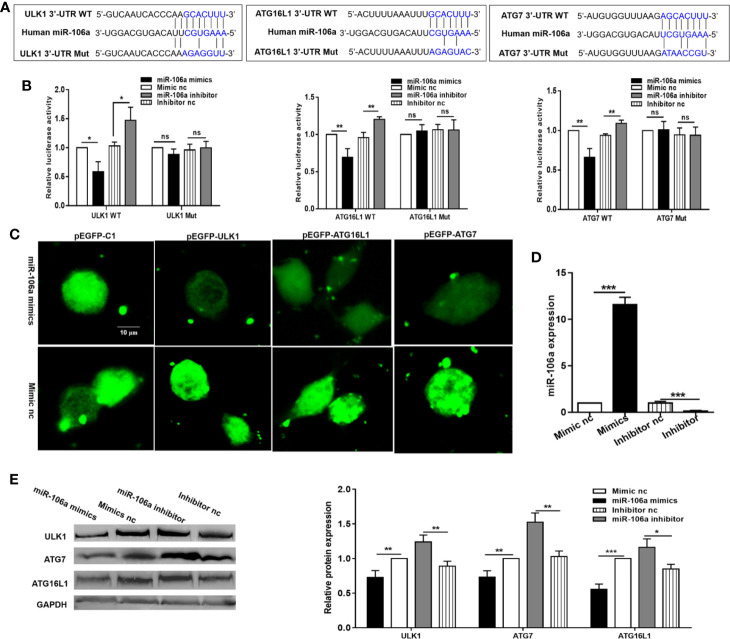
miR-106a directly targets ULK1, ATG7, and ATG16L1. **(A)** Predicted binding between miR-106a and the seed matches in ULK1, ATG7 and ATG16L1 3′-UTRs. The sequence of the ULK1, ATG7, and ATG16L1 3′-UTR seed mutants used for the reporter assays. **(B)** miR-106a regulates ULK1, ATG7, and ATG16L1 3′-UTR reporters. Luciferase reporter assays 24 h after transfection with indicated pmirGLO dual-luciferase reporter vector, co-transfected with miR-106a mimics, miR-106a inhibitor or relevant negative controls (nc). **(C)** Representative fluorescent microscopic image confirm that GFP expression of the pEGFP-ULK1, pEGFP-ATG16L1 and pEGFP-ATG7 reporters was inhibited by miR-106a. HEK-293 cells were co-transfected with the GFP reporter vectors and compared with cells transfected with a mimic or control of miR-106a. Scale bars: 10 μm. **(D)** The THP-1 macrophages were transfected with miR-106a mimics, mimic nc, miR-106a inhibitor or inhibitor nc. The expression levels of miR-106a were measured by qRT-PCR. **(E)** miR-106a decreases ULK1, ATG7 and ATG16L1 protein levels. Western blot analysis 24 h after transfection with miR-106a mimics, mimic nc, miR-106a inhibitor or inhibitor nc. The ULK1, ATG7 and ATG16L1 bands were quantified relative to glyceraldehyde 3-phosphate dehydrogenase (GAPDH). Data represent the means ± SD from at least three independent experiments. *p < 0.05, **p < 0.01, ***p < 0.001.

To establish a direct molecular link, we next examined the ability of miR-106a to regulate ULK1, ATG7, and ATG16L1. As shown in **Figure 2A**, ULK1 holds a single 9mer seed match to miR-106a within the 3′-UTR while ATG16L1 and ATG7 contain a 7mer site and an 8mer site respectively. Moreover, more than three point mutations were introduced into the predicted miR-106a binding motifs. Overexpression of miR-106a significantly inhibited luciferase activity driven by the 3′-UTR constructs (WT), while the mutant 3′-UTR constructs (Mut) either abolished or significantly reduced this effect. Additionally, miR-106a inhibitor significantly strengthened luciferase activity in HEK 293T cells expressing the 3′-UTR reporters, whereas mutation of the miR-106a-binding site abrogated this promotion of luciferase activity (**Figure 2B**), confirming that ULK1, ATG7, and ATG16L1 are putative targets of miR-106a. In order to directly address whether miR-106a binds to the 3′-UTR of target mRNAs, we generated three GFP reporter vectors containing the putative miR-106a binding sites within the 3′-UTRs of ULK1, ATG16L1 and ATG7. GFP fluorescence decreased significantly in cells co-transfected with miR-106a mimics and binding site-containing GFP reporter vectors. However, GFP fluorescence did not decrease significantly in cells transfected with mimic nc or with GFP reporters lacking binding sites (**Figure 2C**). Finally, we examined the effect of miR-106a on the endogenous ULK1, ATG7, and ATG16L1 proteins in THP-1 macrophages. High levels of miR-106a were detected in THP-1 macrophages after transfection with the miR-106a mimics. However, transfection with miR-106a inhibitor significantly reduced the expression levels of miR-106a (**Figure 2D**). As evident from **Figure 2E**, overexpression of miR-106a results in a significant decrease in the expression levels of ULK1, ATG7, and ATG16L1. However, miR-106a inhibitor results in an obvious upregulation of these proteins.

### miR-106a Inhibits Induction of Autophagy in *Mycobacterium tuberculosis*-Infected Macrophages by Targeting ULK1, ATG7, and ATG16L1

**Figure 3:**
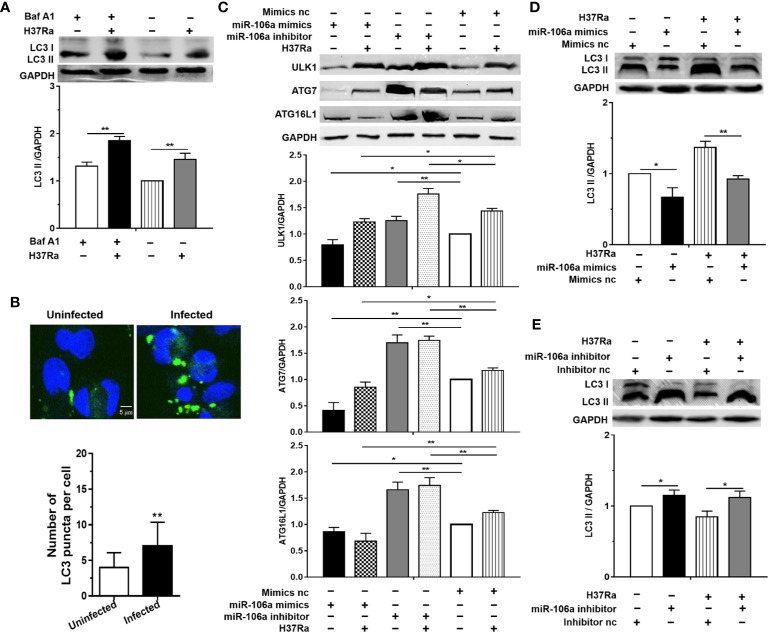
miR-106a inhibits autophagy induction in macrophages by targeting ULK1, ATG7 and ATG16L1. **(A)** THP-1 macrophages were treated with Baf A1 (100 nM) for 2 h, and then were uninfected or infected with H37Ra. LC3-II expression was determined by Western blot, normalized to GAPDH expression. **(B)** THP-1 macrophages were uninfected or infected with H37Ra for 24 h. The cells were fixed and incubated with rabbit anti-LC3 antibody, and stained with goat anti-rabbit IgG (Alexa Fluor 488; green) to detect LC3 puncta by confocal microscopy. Scale bars: 5 μm. The number of LC3 puncta in each cell was also counted. (Uninfected, n = 20; Infected, n = 20). Experiments performed in triplicate. **p < 0.01. **(C)** THP-1 macrophages were transfected with an miR-106a mimic or mimic nc; miR-106a inhibitor or inhibitor nc, and then infected with H37Ra for 24 h. ULK1, ATG7 and ATG16L1 protein levels were determined by Western blot, normalized to GAPDH expression. **(D, E)** The ratio of LC3-II to LC3-I were also determined by Western blot, normalized to GAPDH expression. Data represent the means ± SD from at least three independent experiments. *p < 0.05, **p < 0.01.

To identify whether autophagy could be induced during *M. tuberculosis* infection, the LC3-II expression was investigated, which is considered to be an accurate indicator for autophagosome formation (23, 24). The result showed that there was a marked increase in LC3-II expression with H37Ra infection compared with an uninfected control (**Figure 3A**). In addition, bafilomycin A1 (Baf-A1) challenge led to further accumulation of LC3-II in THP-1 macrophages after H37Ra infection (**Figure 3A**), indicating that H37Ra infection promote autophagic processes. To further confirm that *M. tuberculosis* induce autophagy in THP-1 macrophages, the LC3-II puncta formation was detected by confocal microscopy. H37Ra-infected THP-1 macrophages displayed a significant increase in the number of LC3 puncta compared with uninfected THP-1 macrophages (**Figure 1B**). These results suggest that a complete autophagic response is induced after THP-1 macrophages were infected with H37Ra. To further explore whether miR-106a decreases endogenous ULK1, ATG7, and ATG16L1 during mycobacterial infection, H37Ra-infected THP-1 macrophages were transfected with miR-106a mimic or inhibitor, and protein levels of ULK1, ATG7, and ATG16L1 were measured by Western blot. As shown in **Figure 3C**, miR-106a overexpression decreased the protein levels of ULK1, ATG7, and ATG16L1 in uninfected and H37Ra-infected THP-1 macrophages. In contrast, these protein levels were significantly increased in uninfected and H37Ra-infected THP-1 macrophages, after endogenous miR-106a was blocked by the transfection of a miR-106a inhibitor. To test the hypothesis that miR-106a regulated autophagy in macrophages during *M. tuberculosis* infection, we tested the expression of LC3 by Western blot and counted LC3 puncta by fluorescence microscopy. Western blot results showed that transfection with miR-106a mimics decreased, whereas transfection with miR-106a inhibitor increased, the LC3-II expression in THP-1 macrophages before and after H37Ra infection (**Figures 3D, E****)**.

**Figure 4:**
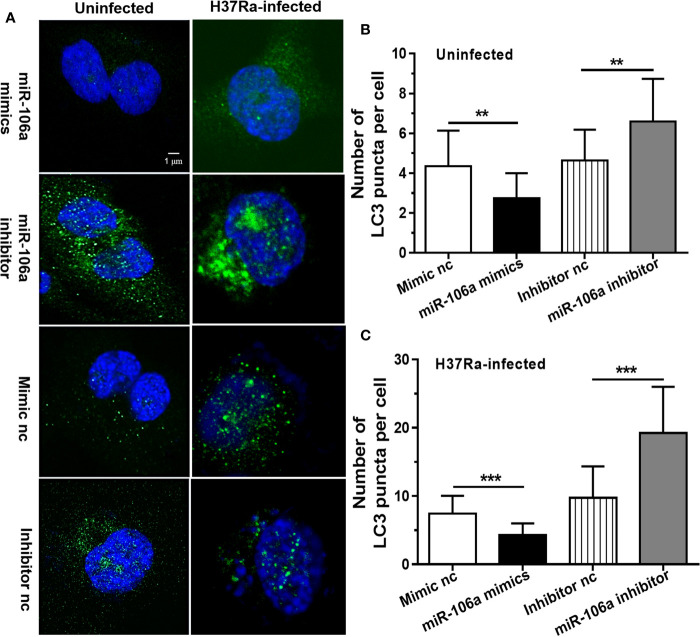
miR-106a mimics significantly decreased the number of LC3 puncta in macrophages. **(A)** THP-1 macrophages were transfected with miR-106a mimic or inhibitor, and then treated with H37Ra for 24 h. The THP-1 macrophages were fixed and incubated with rabbit anti-LC3 antibody, and stained with goat anti-rabbit IgG (Alexa Fluor 488; green) to detect LC3 puncta by confocal microscopy (left, uninfected; right, infected). Scale bars: 1 μm. **(B, C)** Quantitative data of LC3 puncta analysis. (Mimic nc, n = 20; Inhibitor nc, n = 20; Mimic, n = 20; Inhibitor, n = 20). Data represent the means ± SD from three independent experiments. **p < 0.01, ***p < 0.001.

**Figure 5:**
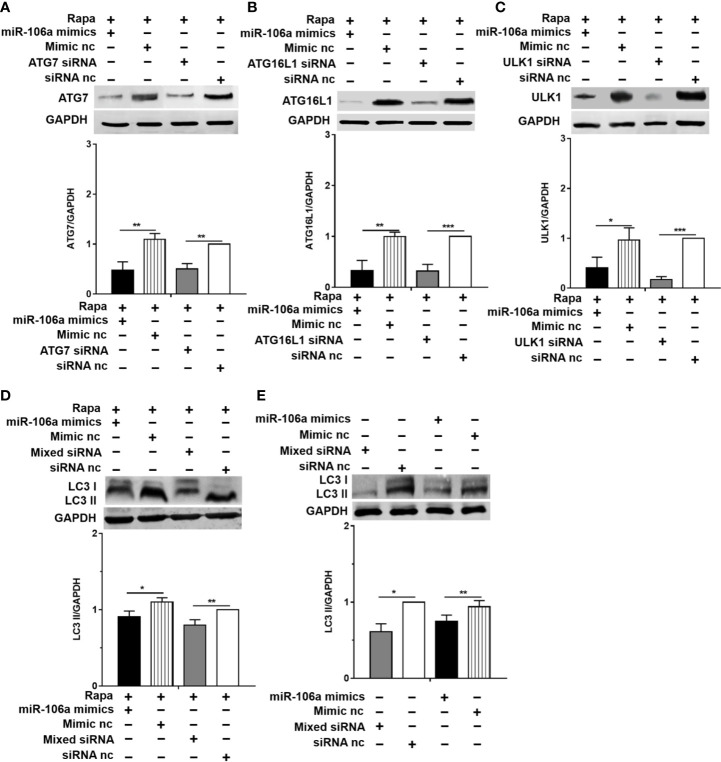
miR-106a and the siRNAs of ATG7, ATG16L1 and ULK1 can inhibit the expression of LC3-II. **(A–C)** The THP-1 macrophages were treated with 50 μg/ml rapamycin for 2 h, and then transfected with miR-106a mimics, ATG7 siRNA, ATG16L1 siRNA or ULK1 siRNA for 24 h. After that, the protein levels of ATG16L1, ATG7 and ULK1 was determined by Western blot. **(D)** The THP-1 macrophages were treated with 50 μg/ml rapamycin for 2 h, and then transfected with miR-106a mimics, or mixture of ATG7 siRNA, ATG16L1 siRNA and ULK1 siRNA for 24 h. After that, LC3-II expression was determined by Western blot. **(E)** The THP-1 macrophages were transfected with miR-106a mimics, or mixture of ATG7 siRNA, ATG16L1 siRNA and ULK1 siRNA for 24 h. After that, LC3-II expression was determined by Western blot. Data represent the means ± SD from three independent experiments. *p < 0.05, **p < 0.01, ***p < 0.001.

**Figure 6:**
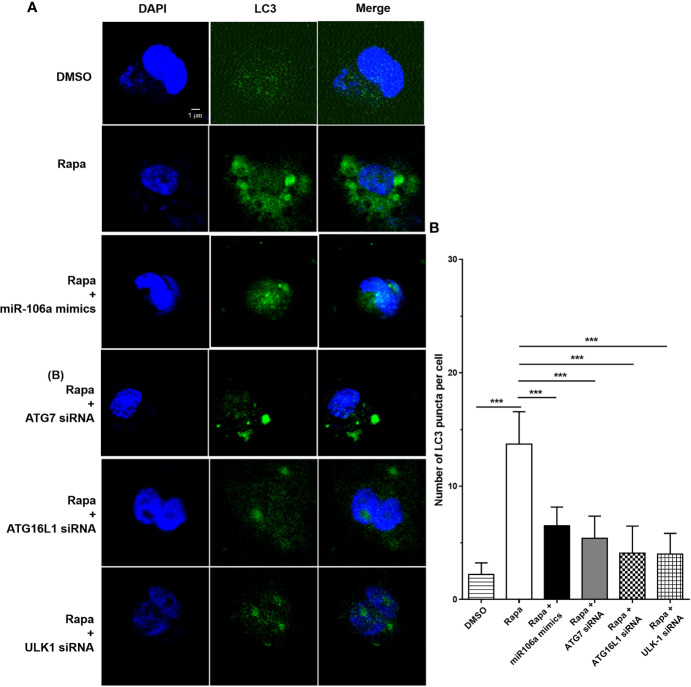
The siRNAs of ATG7, ATG16L1 and ULK1 inhibited autophagosome formation. **(A)** The THP-1 macrophages were treated with 50 μg/ml rapamycin for 2 h, and then transfected with ATG7 siRNA, ATG16L1 siRNA, ULK1 siRNA or miR-106a mimics for 24 h. After that, the THP-1 macrophages were fixed and incubated with Rabbit Anti-LC3 antibody, followed by Alexa Fluor 488-conjugated goat anti-rabbit IgG. LC3 puncta formation was then detected by confocal microscopy. **(B)** Quantitative data of LC3 puncta analysis. (Rapa, n = 10; Rapa plus miR-106a mimics, n = 10; Rapa plus ATG7 siRNA, n = 10; Rapa plus ATG16L1 siRNA, n = 10; Rapa plus ULK1 siRNA, n = 10). Data represent the means ± SD from three independent experiments. ***p < 0.001.

The results of confocal microscopy indicated that miR-106a mimics significantly decreased the number of LC3 puncta in THP-1 macrophages (**Figures 4A–C**). Conversely, the number of LC3 puncta was significantly increased in uninfected and H37Ra-infected THP-1 macrophages after transfection with miR-106a inhibitor, compared with the control condition (**Figures 4A–C**). Transfection with miR-106a mimics, ATG7 siRNA, ATG16L1 siRNA or ULK1 siRNA significantly reduced the protein expression levels of ATG7, ATG16L1, and ULK1 (**Figures 5A–C**), the LC3-II expression (**Figures 5D, E**) and the number of LC3 puncta (**Figures 6A, B**), in THP-1 macrophages with rapamycin, indicating that the siRNAs of ATG7, ATG16L1, and ULK1, and miR-106a mimics can inhibit autophagy. Collectively, these results indicate that miR-106a inhibit autophagy in macrophages.

### TEM Confirms Repression of Autophagy by miR-106a

**Figure 7:**
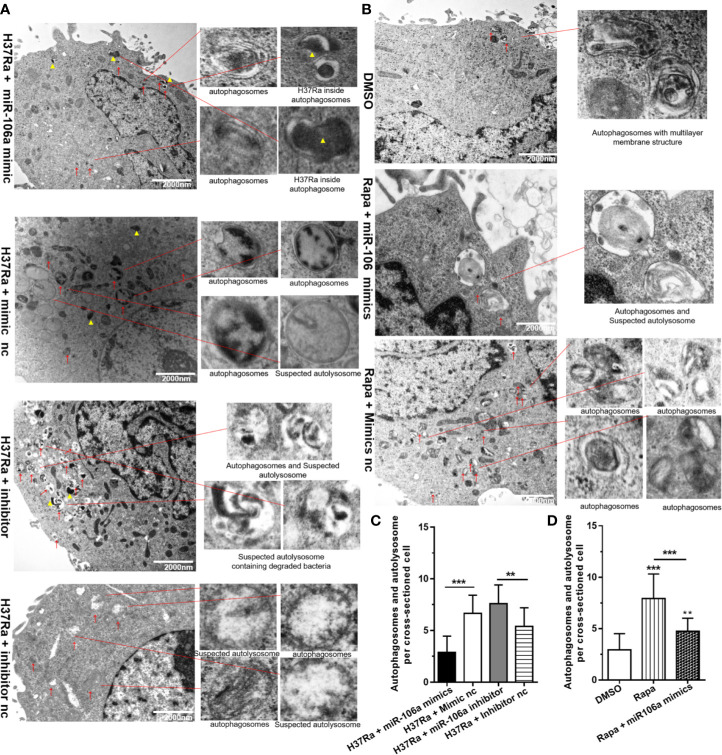
The inhibitory effect on autophagy by miR-106a was confirmed by TEM detection. **(A)** The THP-1 macrophages were transfected with miR-106a mimic or miR-106a inhibitor, and then infected with H37Ra for 24 h. Representative images of TEM. Scale bars represent 2 μm. Autophagosomes or suspected autolysosomes denoted by red arrow heads. H37Ra indicated by yellow triangle. **(B)** The THP-1 macrophages were treated with 50 μg/ml rapamycin for 2 h, and then transfected with miR-106a mimics for 24 h. Representative images of TEM. Scale bars represent 2 μm. Autophagosomes or suspected autolysosomes denoted by red arrow heads. **(C, D)** The number of autophagosomes per cross-sectioned cell was counted (n = 15). Data represent the means ± SD from three independent experiments. **p < 0.01, ***p < 0.001.

In order to gain insight into the regulation effect of miR-106a on autophagy during mycobacterial infection, we perform Transmission Electron Microscopy (TEM) to detected and quantified autophagosomes and autolysosomes. Notably, TEM images revealed an accumulation of numerous autophagosomes and autolysosomes in the cytoplasm of H37Ra-infected THP-1 macrophages transfected with miR-106a inhibitor. However, miR-106a mimics decreased the number of autophagosomes and autolysosomes, confirming our TEM analysis (**Figures 7A, C**). Moreover, transfection with miR-106a mimics could decrease the number of autophagosomes and autolysosomes per cellular cross-section in rapamycin-treated cells (**Figures 7B, D**).

### miR-106a Promotes H37Ra Survival in Macrophages by Inhibiting Autophagy

**Figure 8:**
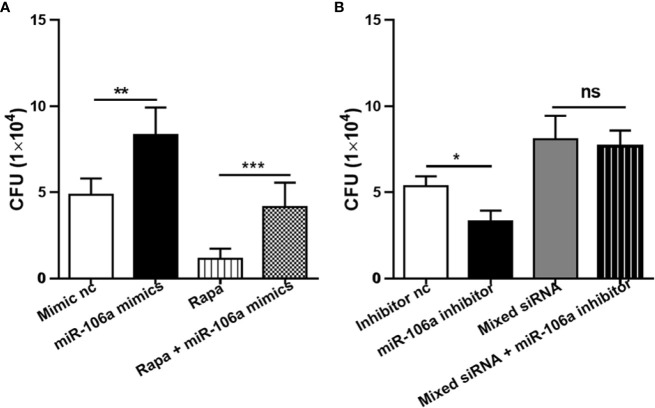
Intracellular survival of H37Ra analyzed by counting CFU. **(A)** The THP-1 macrophages were treated with mimic nc, miR-106a mimics, rapamycin (50 μg/ml) or rapamycin plus miR-106a mimics for 24 h. After infection with H37Ra at a MOI of 10 for 3h, the cells were washed to remove extracellular bacteria, and cultured for an additional 24 h. The cells were lysed and mycobacterial viability (CFU) determined. **p < 0.01, ***p < 0.001. **(B)** The THP-1 macrophages were treated with inhibitor nc, miR-106a inhibitor, mixed siRNA or mixed siRNA plus miR-106a inhibitor for 24 h. After infection with H37Ra at a MOI of 10 for 3h, the cells were washed to remove extracellular bacteria, and cultured for an additional 24 h. The cells were lysed and mycobacterial viability (CFU) determined. *p < 0.05.

The effects of miR-106a on intracellular survival of *M. tuberculosis* in human THP-1 macrophages were analyzed by colony-forming unit (CFU) assay. Importantly, miR-106a mimics promoted (**Figure 8A**), whereas miR-106a inhibitor decreased (**Figure 8B**), intracellular H37Ra growth, compared with the corresponding control conditions. These results support the hypothesis that miR-106a facilitates intracellular survival of H37Ra in macrophages. Moreover, transfection with miR-106a mimics plus rapamycin could promote H37Ra survival compared to treatment with rapamycin in H37Ra-infected THP-1 macrophages (**Figure 8A**), indicating that miR-106a can inhibit rapamycin-induced autophagy. In addition, transfection with mixed siRNA plus miR-106a inhibitor could not inhibit H37Ra survival compared to treatment with mixed siRNA (**Figure 8B**), indicating that miR-106a inhibitor can decrease *M. tuberculosis* CFU *via* autophagy.

## Discussion

Increasing evidence has demonstrated that autophagy plays an essential role in the host innate immune responses against mycobacterial infection (25, 26). However, the molecular mechanism of autophagy-mediated mycobacterial clearance remains unclear. There is growing evidence that miRNAs are regulators of genes involved in many aspects of immune system function, including differentiation of immune cells (27) and regulation of the host immune defense mechanisms against microbial infection (28). However, the immune regulatory functions of miRNAs in autophagy-mediated mycobacterial clearance, especially miR-17 family miRNAs, need to be further explored. In this study, we describe a novel role of miR-106a in modulating autophagy process and mycobacterial elimination in human macrophages by targeting ULK1, ATG7, and ATG16L1, which may provide a better understanding of the host innate immune responses against *M. tuberculosis*.

miR-106a is a member of miR-17 family miRNAs, which are broadly conserved and involved in a variety of biological pathways (29, 30). Evidence is mounting that miR-106a plays key regulatory roles in autophagy, especially in cancer. For instance, miR-106a inhibits tumor cell death in colorectal cancer by targeting ATG7 (31). miR-106a suppresses ULK1 expression and thereby sensitizes lung cancer cells to Src-TKI treatment (32). Moreover, miR-106a targets the important autophagy gene ULK1 in acute myeloid leukemia cells (33). Most importantly, a research has shown that miR-106a regulates macrophage inflammatory responses by targeting SIRPa, indicating a potential role of miR-106a in the host immune response (34). However, the exact role of miR-106a in human macrophages during *M. tuberculosis* infection remains largely unclear. Indeed, our study showed that miR-106a functioned as a negative regulator in autophagy responses during *M. tuberculosis* infection, and inhibition of miR-106a expression promoted autophagy process to facilitate mycobacterial clearance.

Furthermore, we investigated the molecular mechanism by which miR-106a regulates autophagy responses in mycobacterial infected macrophages. We identified that ULK1, ATG16L1 and ATG7 are targets for miR-106a. ULK1, ATG7, and ATG16L1 are essential for autophagy. ULK1 can function in a complex with at least three protein partners: FIP200, ATG13 and ATG101. The formation of the autophagosome is mediated by the ULK1 complex (14). ULK1-deficient cells increased *M. tuberculosis* replication, and decreased selective autophagy (35). ATG7 is a master regulators of autophagy process, which is involved in autophagosome formation and vesicle progression (15). ATG7 knockout mice displayed increased susceptibility to *Klebsiella pneumoniae* infection, with decreased survival rates, increased bacterial burdens, and intensified lung injury (36). Moreover, ATG7-deficient macrophages exhibited enhanced mycobacterial uptake and growth by modulating the expression of scavenger receptors, and ATG7 knockout mice exhibited increased susceptibility to mycobacterial infection (37). ATG16L1 takes part in the elongation of the autophagosomal membrane (16). It has been reported that ATG16L1 conditional knockout mice exhibit defective autophagy and are more susceptible to *Salmonella* infection (38). Our study indicates that miR-106a downregulates ULK1, ATG7, and ATG16L1 proteins, thus inhibiting autophagy process in human macrophages.

Autophagy is a crucial defense immune response after the encounter of intracellular bacterial infection, including *M. tuberculosis* (39). Major steps in the process of autophagy contain initiation, nucleation, elongation, and autophagosome maturation as well as fusion of autophagosomes with lysosomes (40). Recent studies provide evidence that several miRNAs modulate autophagy process during mycobacterial infection (41). For example, miR-125a inhibits autophagy process and antimicrobial responses through targeting UVRAG during mycobacterial infection (42). miR-155 accelerated the autophagic response to eliminate intracellular Mycobacteria by targeting Rheb in macrophages (43). However, miR-155 subverts autophagy by targeting ATG3 in human dendritic cells (44). It is reported that miR-17-5p regulates autophagy in *M. tuberculosis*-infected macrophages by targeting Mcl-1 and STAT3 (22). miR-27a promotes the intracellular survival of *M. tuberculosis* by regulating Ca^2+^-associated autophagy (45). miR-144-5p inhibits antibacterial autophagy and the innate host immune response against *M. tuberculosis* in human monocytes and macrophages by targeting DRAM2 (46). *M. tuberculosis* can inhibit integrated pathways involved in autophagy to support bacterial intracellular survival and persistence by inducing miR-33 and miR-33* (47). Another study showed that miR-20a inhibits autophagic response and favors BCG survival in murine macrophages by targeting ATG7 and ATG16L1 (48). In the present study, miR-106a decreased in differentiated THP-1 macrophages after H37Ra infection. Functional assays demonstrated that inhibition of miR-106a promoted the processing of LC3 and the accumulation of LC3 puncta in uninfected and H37Ra-infected THP-1 macrophages, which indicates that functioned as a negative regulator of autophagy during mycobacterial infection. The inhibitory effect of miR-106a on autophagy during mycobacterial infection was also confirmed by TEM observation. In addition, we found that inhibited expression of miR-106a decreased the intracellular survival of H37Ra, whereas mimics of miR-106a increased mycobacterial survival, indicating that downregulation of miR-106a promoted autophagy process as a novel mechanism for host defense against *M. tuberculosis* infection.

**Figure 9:**
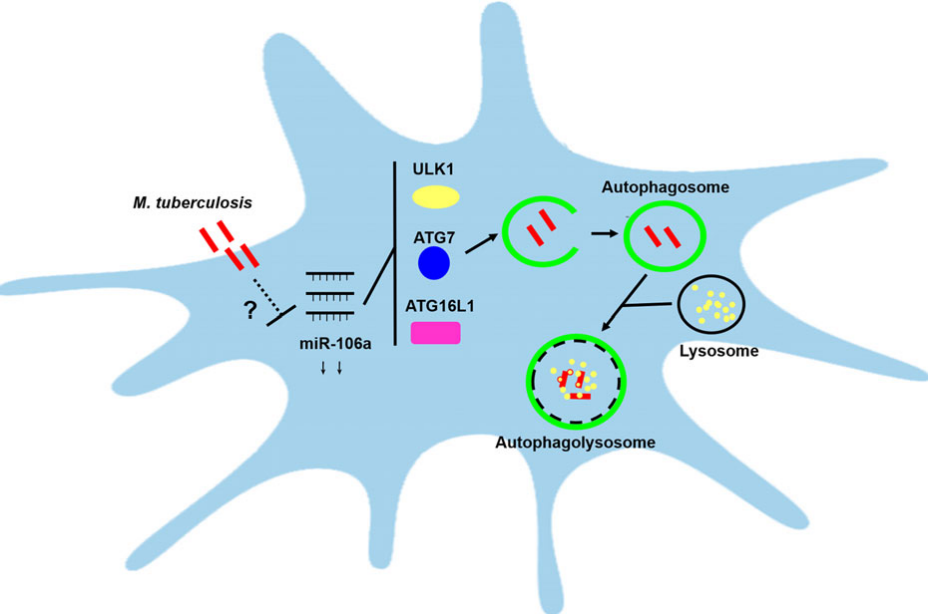
Schematic diagram of miR-106a regulating autophagy by targeting ULK1, ATG7 and ATG16L1. miR-106a expression was downregulated in human macrophages after mycobacterial infection, however it remains unclear about the mechanisms by which miR-106a is reduced. miR-106a can perform the regulation of autophagy and antimicrobial responses by targeting ULK1, ATG7 and ATG16L1.

Overall, our study reveals a novel pathway through which host can promote autophagic response to facilitate mycobacterial clearance by reducing miR-106a. Furthermore, miR-106a performs the regulation of autophagy by targeting ULK1, ATG16L1 and ATG7 after mycobacterial infection (**Figure 9**). This study reveals a previously unrecognized role of miR-106a in autophagy regulation during mycobacterial infection, which may provide a potential target for diagnosis and treatments of tuberculosis.

## Data Availability Statement

The datasets presented in this study can be found in online repositories. The names of the repository/repositories and accession number(s) can be found in the article/supplementary material.

## Author Contributions

LG and KL conceived and designed the experiments. DH, FZ, XL, and MH performed the experiments. DH, FZ, and ZJ analyzed the data. XH, GZ, XH, WA, and GX contributed the reagents/materials/analysis tools. LG, KL, and NS wrote the manuscript. All authors contributed to the article and approved the submitted version.

## Funding

This work was supported by the National Natural Science Foundation of China (81760359, 32070930, and 31660267), Key R & D Plan Project of Ningxia Autonomous Region (2020BFG02012), Natural Science Foundation of Ningxia (2020AAC03152, 2019AAC03079, 2018AAC02018 and 2018AAC03074), Science Research Project of Ningxia’s Colleges (NGY2020043 and NGY2018-75), Preponderant Discipline Construction Project of Ningxia Medical University (NGY2020043 and NGY2018-75), Science and Technology Project of Jiangsu Market Supervision Administration (KJ207561), First-Class Discipline Construction Founded Project of Ningxia Medical University and the School of Clinical Medicine (NXYLXK2017A05), Young Scientific and Technological Talents of Ningxia (TJGC2018079), Ningxia Youth Top Talent training Project and “Light of the West” Talent Training Programme of the Chinese Academy of Sciences.

## Conflict of Interest

The authors declare that the research was conducted in the absence of any commercial or financial relationships that could be construed as a potential conflict of interest.

## References

[1] World Health Organization Global tuberculosis report 2020. *World Health Organization* (2020). Available at: https://apps.who.int/iris/handle/10665/336069. License: CC BY-NC-SA 3.0 IGO.

[2] Banuls AL Sanou A Anh NT Godreuil S *Mycobacterium tuberculosis*: ecology and evolution of a human bacterium. J Med Microbiol (2015) 64(11):1261–9. 10.1099/jmm.0.000171 26385049

[3] Getahun H Matteelli A Chaisson RE Raviglione M Latent *Mycobacterium tuberculosis* infection. N Engl J Med (2015) 372(22):2127–35. 10.1056/NEJMra1405427 26017823

[4] Philips JA Ernst JD Tuberculosis pathogenesis and immunity. Annu Rev Pathol (2012) 7:353–84. 10.1146/annurev-pathol-011811-132458 22054143

[5] Franco LH Nair VR Scharn CR Xavier RJ Torrealba JR Shiloh MU The Ubiquitin Ligase Smurf1 Functions in Selective Autophagy of *Mycobacterium tuberculosis* and Anti-tuberculous Host Defense. Cell Host Microbe (2017) 21(1):59–72. 10.1016/j.chom.2016.11.002 28017659 PMC5699477

[6] Saiga H Shimada Y Takeda K Innate immune effectors in mycobacterial infection. Clin Dev Immunol (2011) 2011:347594. 10.1155/2011/347594 21274449 PMC3025378

[7] Sia JK Georgieva M Rengarajan J Innate Immune Defenses in Human Tuberculosis: An Overview of the Interactions between *Mycobacterium tuberculosis* and Innate Immune Cells. J Immunol Res (2015) 2015:747543. 10.1155/2015/747543 26258152 PMC4516846

[8] Liu CH Liu H Ge B Innate immunity in tuberculosis: host defense vs pathogen evasion. Cell Mol Immunol (2017) 14(12):963–75. 10.1038/cmi.2017.88 PMC5719146 28890547

[9] Baena A Porcelli SA Evasion and subversion of antigen presentation by *Mycobacterium tuberculosis*. Tissue Antigens (2009) 74(3):189–204. 10.1111/j.1399-0039.2009.01301.x 19563525 PMC2753606

[10] Stanley SA Cox JS Host-pathogen interactions during *Mycobacterium tuberculosis* infections. Curr Top Microbiol Immunol (2013) 374:211–41. 10.1007/82_2013_332 23881288

[11] Deretic V Saitoh T Akira S Autophagy in infection, inflammation and immunity. Nat Rev Immunol (2013) 13(10):722–37. 10.1038/nri3532 PMC5340150 24064518

[12] Xie Y Kang R Sun X Zhong M Huang J Klionsky DJ Posttranslational modification of autophagy-related proteins in macroautophagy. Autophagy (2015) 11(1):28–45. 10.4161/15548627.2014.984267 25484070 PMC4502723

[13] Singh R Cuervo AM Autophagy in the cellular energetic balance. Cell Metab (2011) 13(5):495–504. 10.1016/j.cmet.2011.04.004 21531332 PMC3099265

[14] Zachari M Ganley IG The mammalian ULK1 complex and autophagy initiation. Essays Biochem (2017) 61(6):585–96. 10.1042/EBC20170021 PMC5869855 29233870

[15] Pattison JS Osinska H Robbins J Atg7 induces basal autophagy and rescues autophagic deficiency in CryABR120G cardiomyocytes. Circ Res (2011) 109(2):151–60. 10.1161/CIRCRESAHA.110.237339 PMC3150753 21617129

[16] Archna A Scrima A Identification, biochemical characterization and crystallization of the central region of human ATG16L1. Acta Crystallogr F Struct Biol Commun (2017) 73(Pt 10):560–7. 10.1107/S2053230X17013280 PMC5633923 28994404

[17] Hammond SM An overview of microRNAs. Adv Drug Delivery Rev (2015) 87:3–14. 10.1016/j.addr.2015.05.001 PMC4504744 25979468

[18] Shukla GC Singh J Barik S MicroRNAs: Processing, Maturation, Target Recognition and Regulatory Functions. Mol Cell Pharmacol (2011) 3(3):83–92. 22468167 PMC3315687

[19] Staedel C Darfeuille F MicroRNAs and bacterial infection. Cell Microbiol (2013) 15(9):1496–507. 10.1111/cmi.12159 23795564

[20] Long J He Q Yin Y Lei X Li Z Zhu W The effect of miRNA and autophagy on colorectal cancer. Cell Prolif (2020) 53(10):e12900. 10.1111/cpr.12900 32914514 PMC7574865

[21] Gozuacik D Akkoc Y Ozturk DG Kocak M Autophagy-Regulating microRNAs and Cancer. Front Oncol (2017) 7:65. 10.3389/fonc.2017.00065 28459042 PMC5394422

[22] Kumar R Sahu SK Kumar M Jana K Gupta P Gupta UD MicroRNA 17-5p regulates autophagy in *Mycobacterium tuberculosis*-infected macrophages by targeting Mcl-1 and STAT3. Cell Microbiol (2016) 18(5):679–91. 10.1111/cmi.12540 26513648

[23] Klionsky DJ Cuervo AM Seglen PO Methods for monitoring autophagy from yeast to human. Autophagy (2007) 3(3):181–206. 10.4161/auto.3678 17224625

[24] Yoshii SR Mizushima N Monitoring and Measuring Autophagy. Int J Mol Sci (2017) 18(9):1865. 10.3390/ijms18091865 PMC5618514 28846632

[25] Wang J Wang R Wang H Yang X Yang J Xiong W Glucocorticoids Suppress Antimicrobial Autophagy and Nitric Oxide Production and Facilitate Mycobacterial Survival in Macrophages. Sci Rep (2017) 7(1):982. 10.1038/s41598-017-01174-9 28428627 PMC5430514

[26] Bah A Lacarriere C Vergne I Autophagy-Related Proteins Target Ubiquitin-Free Mycobacterial Compartment to Promote Killing in Macrophages. Front Cell Infect Microbiol (2016) 6:53. 10.3389/fcimb.2016.00053 27242971 PMC4863073

[27] Jeker LT Bluestone JA MicroRNA regulation of T-cell differentiation and function. Immunol Rev (2013) 253(1):65–81. 10.1111/imr.12061 23550639 PMC3621017

[28] Tang B Li N Gu J Zhuang Y Li Q Wang HG Compromised autophagy by MIR30B benefits the intracellular survival of Helicobacter pylori. Autophagy (2012) 8(7):1045–57. 10.4161/auto.20159 PMC3429542 22647547

[29] Fuziwara CS Kimura ET Insights into Regulation of the miR-17-92 Cluster of miRNAs in Cancer. Front Med (Lausanne) (2015) 2:64. 10.3389/fmed.2015.00064 26442266 PMC4561802

[30] Gruszka R Zakrzewska M The Oncogenic Relevance of miR-17-92 Cluster and Its Paralogous miR-106b-25 and miR-106a-363 Clusters in Brain Tumors. Int J Mol Sci (2018) 19(3):879. 10.3390/ijms19030879 PMC5877740 29547527

[31] Hao H Xia G Wang C Zhong F Liu L Zhang D miR-106a suppresses tumor cells death in colorectal cancer through targeting ATG7. Med Mol Morphol (2017) 50(2):76–85. 10.1007/s00795-016-0150-7 27981410

[32] Rothschild S II Gautschi O Batliner J Gugger M Fey MF Tschan MP MicroRNA-106a targets autophagy and enhances sensitivity of lung cancer cells to Src inhibitors. Lung Cancer (2017) 107:73–83. 10.1016/j.lungcan.2016.06.004 27372519

[33] Jin J Britschgi A Schlafli AM Humbert M Shan-Krauer D Batliner J Low Autophagy (ATG) Gene Expression Is Associated with an Immature AML Blast Cell Phenotype and Can Be Restored during AML Differentiation Therapy. Oxid Med Cell Longev (2018) 2018:1482795. 10.1155/2018/1482795 29743969 PMC5878891

[34] Zhu D Pan C Li L Bian Z Lv Z Shi L MicroRNA-17/20a/106a modulate macrophage inflammatory responses through targeting signal-regulatory protein alpha. J Allergy Clin Immunol (2013) 132(2):426–36 e8. 10.1016/j.jaci.2013.02.005 23562609 PMC5882493

[35] Horne DJ Graustein AD Shah JA Peterson G Savlov M Steele S Human ULK1 Variation and Susceptibility to *Mycobacterium tuberculosis* Infection. J Infect Dis (2016) 214(8):1260–7. 10.1093/infdis/jiw347 PMC5034956 27485354

[36] Ye Y Li X Wang W Ouedraogo KC Li Y Gan C Atg7 deficiency impairs host defense against Klebsiella pneumoniae by impacting bacterial clearance, survival and inflammatory responses in mice. Am J Physiol Lung Cell Mol Physiol (2014) 307(5):L355–63. 10.1152/ajplung.00046.2014 PMC4154251 24993132

[37] Bonilla DL Bhattacharya A Sha Y Xu Y Xiang Q Kan A Autophagy regulates phagocytosis by modulating the expression of scavenger receptors. Immunity (2013) 39(3):537–47. 10.1016/j.immuni.2013.08.026 PMC8059138 24035364

[38] Conway KL Kuballa P Song JH Patel KK Castoreno AB Yilmaz OH Atg16l1 is required for autophagy in intestinal epithelial cells and protection of mice from Salmonella infection. Gastroenterology (2013) 145(6):1347–57. 10.1053/j.gastro.2013.08.035 PMC3840157 23973919

[39] Winchell CG Steele S Kawula T Voth DE Dining in: intracellular bacterial pathogen interplay with autophagy. Curr Opin Microbiol (2016) 29:9–14. 10.1016/j.mib.2015.09.004 26462048 PMC4755823

[40] Nakamura S Yoshimori T New insights into autophagosome-lysosome fusion. J Cell Sci (2017) 130(7):1209–16. 10.1242/jcs.196352 28302910

[41] Kim JK Kim TS Basu J Jo EK MicroRNA in innate immunity and autophagy during mycobacterial infection. Cell Microbiol (2017) 19(1):e12678. 10.1111/cmi.12687 27794209

[42] Kim JK Yuk JM Kim SY Kim TS Jin HS Yang CS MicroRNA-125a Inhibits Autophagy Activation and Antimicrobial Responses during Mycobacterial Infection. J Immunol (2015) 194(11):5355–65. 10.4049/jimmunol.1402557 25917095

[43] Wang J Yang K Zhou L Minhaowu Wu Y Zhu M MicroRNA-155 promotes autophagy to eliminate intracellular mycobacteria by targeting Rheb. PloS Pathog (2013) 9(10):e1003697. 10.1371/journal.ppat.1003697 24130493 PMC3795043

[44] Etna MP Sinigaglia A Grassi A Giacomini E Romagnoli A Pardini M *Mycobacterium tuberculosis*-induced miR-155 subverts autophagy by targeting ATG3 in human dendritic cells. PloS Pathog (2018) 14(1):e1006790. 10.1371/journal.ppat.1006790 29300789 PMC5771628

[45] Liu F Chen J Wang P Li H Zhou Y Liu H MicroRNA-27a controls the intracellular survival of *Mycobacterium tuberculosis* by regulating calcium-associated autophagy. Nat Commun (2018) 9(1):4295. 10.1038/s41467-018-06836-4 30327467 PMC6191460

[46] Kim JK Lee HM Park KS Shin DM Kim TS Kim YS MIR144* inhibits antimicrobial responses against *Mycobacterium tuberculosis* in human monocytes and macrophages by targeting the autophagy protein DRAM2. Autophagy (2017) 13(2):423–41. 10.1080/15548627.2016.1241922 PMC5324854 27764573

[47] Ouimet M Koster S Sakowski E Ramkhelawon B van Solingen C Oldebeken S *Mycobacterium tuberculosis* induces the miR-33 locus to reprogram autophagy and host lipid metabolism. Nat Immunol (2016) 17(6):677–86. 10.1038/ni.3434 PMC4873392 27089382

[48] Guo L Zhao J Qu Y Yin R Gao Q Ding S microRNA-20a Inhibits Autophagic Process by Targeting ATG7 and ATG16L1 and Favors Mycobacterial Survival in Macrophage Cells. Front Cell Infect Microbiol (2016) 6:134. 10.3389/fcimb.2016.00134 27803889 PMC5067373

